# Modeling Post-Scratching Locomotion with Two Rhythm Generators and a Shared Pattern Formation

**DOI:** 10.3390/biology10070663

**Published:** 2021-07-14

**Authors:** Jesus A. Tapia, Argelia Reid, John Reid, Saul M. Dominguez-Nicolas, Elias Manjarrez

**Affiliations:** 1 Facultad de Ciencias Biológicas, Benemérita Universidad Autónoma de Puebla, Blvd, Valsequillo y Av. San Claudio, Ed. BIO 1, Puebla Pue 72570, Mexico; jesus.tapia@correo.buap.mx; 2 Instituto de Fisiología, Benemérita Universidad Autónoma de Puebla, 14 Sur 6301, Col. San Manuel, Apartado Postal 406, Puebla Pue 72570, Mexico; jolieargelie@gmail.com (A.R.); reidjd2@gmail.com (J.R.); 3 Centro de Investigación en Micro y Nanotecnología, Universidad Veracruzana, Calzada Ruiz Cortines 455 Boca del Rio, Veracruz 94294, Mexico; saudominguez@uv.mx

**Keywords:** mathematical model, movement production, movement sequence, CPG, central pattern generator, locomotion, scratching, post-scratching locomotion

## Abstract

**Simple Summary:**

Post-scratching locomotion in cats refers to the spontaneous occurrence of an episode of locomotion generated after an event of scratching. This phenomenon suggests the potential existence of shared neurons in the spinal cord mediating the transition from one rhythmic motor task to another. Here, we examine this possibility with a mathematical model, reproducing the experimental observations. Our findings reveal a possible mechanism in which the central nervous system could share neuronal circuits from two central pattern generators to produce a sequence of different rhythmic motor actions.

**Abstract:**

This study aimed to present a model of post-scratching locomotion with two intermixed central pattern generator (CPG) networks, one for scratching and another for locomotion. We hypothesized that the rhythm generator layers for each CPG are different, with the condition that both CPGs share their supraspinal circuits and their motor outputs at the level of their pattern formation networks. We show that the model reproduces the post-scratching locomotion latency of 6.2 ± 3.5 s, and the mean cycle durations for scratching and post-scratching locomotion of 0.3 ± 0.09 s and 1.7 ± 0.6 s, respectively, which were observed in a previous experimental study. Our findings show how the transition of two rhythmic movements could be mediated by information exchanged between their CPG circuits through routes converging in a common pattern formation layer. This integrated organization may provide flexible and effective connectivity despite the rigidity of the anatomical connections in the spinal cord circuitry.

## 1. Introduction

The question of how a sequence of movements is generated has intrigued biologists for years. Despite investigations during this time, advances have been scarce and have been mainly for behavioral and modeling analysis [1,2,3,4]. This lack of advances is perhaps due to the complex control of many neuronal variables during decision making and visuomotor feedback in the production of movement sequences.

Though these variables appear to impose limitations, an alternative to overcome them is to analyze movement sequences between different rhythmic motor tasks, which are performed even without the brain and with the absence of sensory feedback. Rhythmic motor tasks can be modeled by central pattern generators (CPGs) and experimentally examined in reduced animal preparations, such as turtles and cats, among other animals. Some relevant advances regarding CPG networks have been related to the possible functional architecture of two layers [5]. The two-level CPG model has a typical “rhythm generator layer” that controls the firing of the “pattern formation layer”, which is responsible for the final motoneuron activation in the spinal cord. This neuronal architecture was suggested from the modeling of experimental data of fictive locomotion in decerebrate cats and has served to interpret the physiological nature of rhythmic motor tasks.

Pioneering studies demonstrated that the turtle spinal cord contains shared neurons to coordinate transitions between different rhythmic movements of locomotion and scratching [6] or swimming and scratching [7,8,9,10,11]. Similar results were found in cats [12,13], suggesting that shared CPG neurons could have been conserved during evolution. Studies in cats showed spinal neurons rhythmically active during both scratching and locomotion [12,13]. For instance, spinal neurons mediating the reciprocal Ia inhibition may contribute to motoneuron hyperpolarizations during the inactive phase of scratching and locomotion in cats [12], hence demonstrating the existence of shared spinal neurons in both motor tasks.

A phenomenon that is consistent with the aforementioned studies is post-scratching locomotion in cats [13], which consists of a fictive locomotion episode, produced 6.2 ± 3.5 s after an event of fictive scratching. Spinal neurons firing during scratching and post-scratching locomotion keep their firing phase during the flexor or extensor stages of both rhythmic movements. This finding suggests the existence of shared spinal neurons related to both motor tasks [13]. In this context, the present study aimed to develop a computational model to explain the experimental observations of cycle durations and switching delays in the spontaneous transition from scratching to post-scratching locomotion in the cat. 

We hypothesized that such shared spinal neurons could belong to the pattern formation network located in the two-layer architecture of central pattern generators. We also hypothesized that a descending input to the rhythm generator layers of these CPGs could be associated with the switching from scratching to post-scratching locomotion. Here, we show that the model reproduces the experimental observations [13] and sheds light on the functional organization of spinal circuits active in the generation and transition of two different rhythmic movements.

## 2. Materials and Methods

### Model Equations

**Figure 1:**
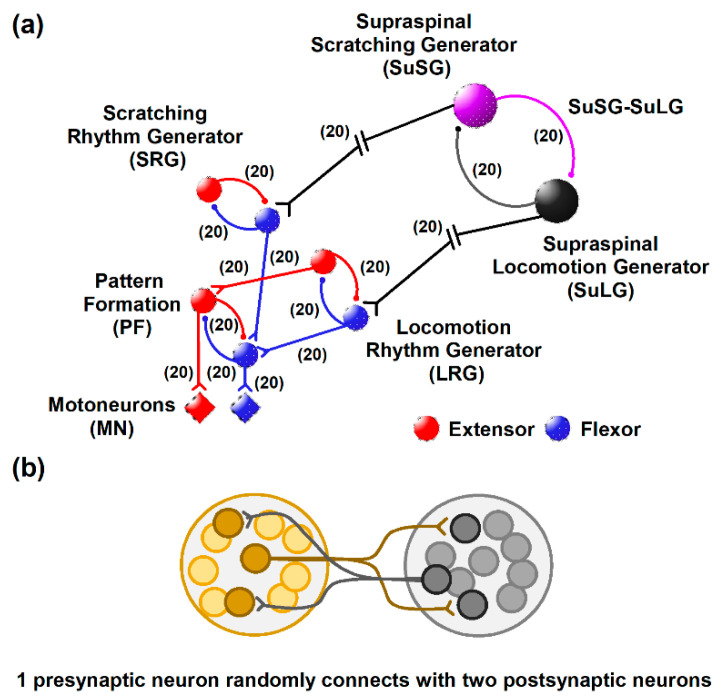
(**a**) Schematics for the proposed post-scratching locomotion model. Supraspinal scratching generator (SuSG), supraspinal locomotion generator (SuLG), scratching rhythm generator (SRG), locomotion rhythm generator (LRG), pattern formation (PF), motoneurons (MNs). Flexor neurons are in blue and extensor neurons are in red. (**b**) The topology of the synaptic connections. The half centers were made of 10 interneurons each, with a sparse divergence of synaptic connections. Specifically, every individual presynaptic neuron in the half centers is randomly connected with 20% of the postsynaptic neurons.

This model is based on a series of modified Morris–Lecar equations [14,15], the synaptic currents equation [16], and the two-layer CPG model [17,18]. The set of differential equations was solved in Matlab using a 4th-order Runge–Kutta method. We constructed the model based on experimental observations of the post-scratching locomotion phenomenon. The model consisted of four hypothetical half centers (Figure 1) as follows. First, a supraspinal half center is composed of a supraspinal scratching generator (SuSG) and a supraspinal locomotion generator (SuLG); this supraspinal half center is labeled as SuSG-SuLG in Figure 1. Second, there is a half center consisting of the scratching rhythm generator (SRG); third, a half center representing the locomotion rhythm generator (LRG); and fourth, a common pattern formation layer that is labeled as PF.

Note that the SuSG-SuLG half center sends asymmetric descending inputs to the SRG and LRG layers, which in turn send symmetric inputs to the common PF layer (Figure 1a). The topology of our model consisted of 80 interneurons and 20 motoneurons, with a total number of 300 synaptic connections. The half centers were made of 10 interneurons each, with a sparse divergence of synaptic connections. Specifically, every individual presynaptic neuron was randomly connected with 20% of the postsynaptic neurons (Figure 1b). We used this small number of neurons and synaptic connections following a previous model for scratching in cats. However, other models for scratching in turtles employ a larger number of neurons in their topology (see the discussion section). The total number of synaptic connections between neuronal groups is shown with numbers in parentheses in Figure 1a.

The SuSG-SuLG half center is responsible for the onset of fictive scratching and subsequent activation of post-scratching locomotion; this half center is mutually connected via a set of inhibitory neurons. The SRG half center consists of two groups of neurons that are mutually inhibited, which receive input from the sum of the SuSG-SuLG half center, and in turn, reproduce frequency and duty cycles of fictive scratching. The LRG half center is responsible for producing locomotor frequency and duty cycles according to experimental observations. Finally, the fourth half center, labeled as PF, is a common pattern formation layer that is activated by either the SRG or LRG layers to subsequently activate the corresponding motoneurons (labeled as MNs) in the common final pathway.

The equations employed for the implementation of this model read as follows:

$$C\frac{dv}{dt}=I_{app}-I_{ionic}-I_{synaptic}$$

$$\frac{dw}{dt}=\varphi \tau \left(v\right)\left(w_{\infty }\left(v\right)-w\right)$$

$$\frac{dCa}{dt}=\varepsilon \left(-\mu g_{Ca}m_{\infty }\left(v\right)\left(v-V_{Ca}\right)-Ca\right)$$

$$I_{ionic}=g_{Ca}m_{\infty }\left(v\right)\left(v-V_{Ca}\right)+g_{K}w\left(v-V_{K}\right)-g_{KCa}z\left(Ca\right)\left(v-V_{K}\right)-g_{L}\left(v-V_{L}\right)$$

$$m_{\infty }=\frac{1}{2}\left(1+\tanh\left(\frac{v+V_{1}}{V_{2}}\right)\right)$$

$$w_{\infty }=\frac{1}{2}\left(1+\tanh\left(\frac{v+W_{1}}{W_{2}}\right)\right)$$

$$\tau \left(v\right)=\cosh\left(\frac{v-W_{1}}{2W_{2}}\right)$$

$$z\left(Ca\right)=\frac{Ca}{Ca_{0}+Ca}$$

$$I_{synaptic}=\underset{j}{\sum }g_{ij}^{syn}r_{j}\left(v_{i}-E_{s}\right)$$

$$r_{j}=1-e^{-\alpha t}\;\;\;\;\mathrm{for}\;t\;<\;t_{\mathrm{on}}$$

$$r_{j}=\left(1-e^{-\alpha t}\right)e^{-\beta \left(t-t_{\mathrm{on}}\right)}\;\;\;\;\mathrm{for}\;t\;>t_{\mathrm{on}}$$

The parameter *t_on_* is the time at which a neuronal membrane potential increased and crossed a −15 mV threshold. At that time, we considered the occurrence of an action potential. All parameters used in this simulation are summarized in Table 1. They were modified during simulations to be consistent with the physiological findings.

## 3. Results

The model illustrated in Figure 1 reproduces the switching between scratching and post-scratching locomotion as in the experimental observations [13]. In the model, this alternation is caused by a fatigue effect in the supraspinal scratching generator (SuSG) that connects with the scratching rhythm generator (SRG) and subsequently with the pattern formation (PF) layer. This fatigue was a result of the modeled neurons that exhibited a prolonged spike frequency adaptation. 

**Figure 2:**
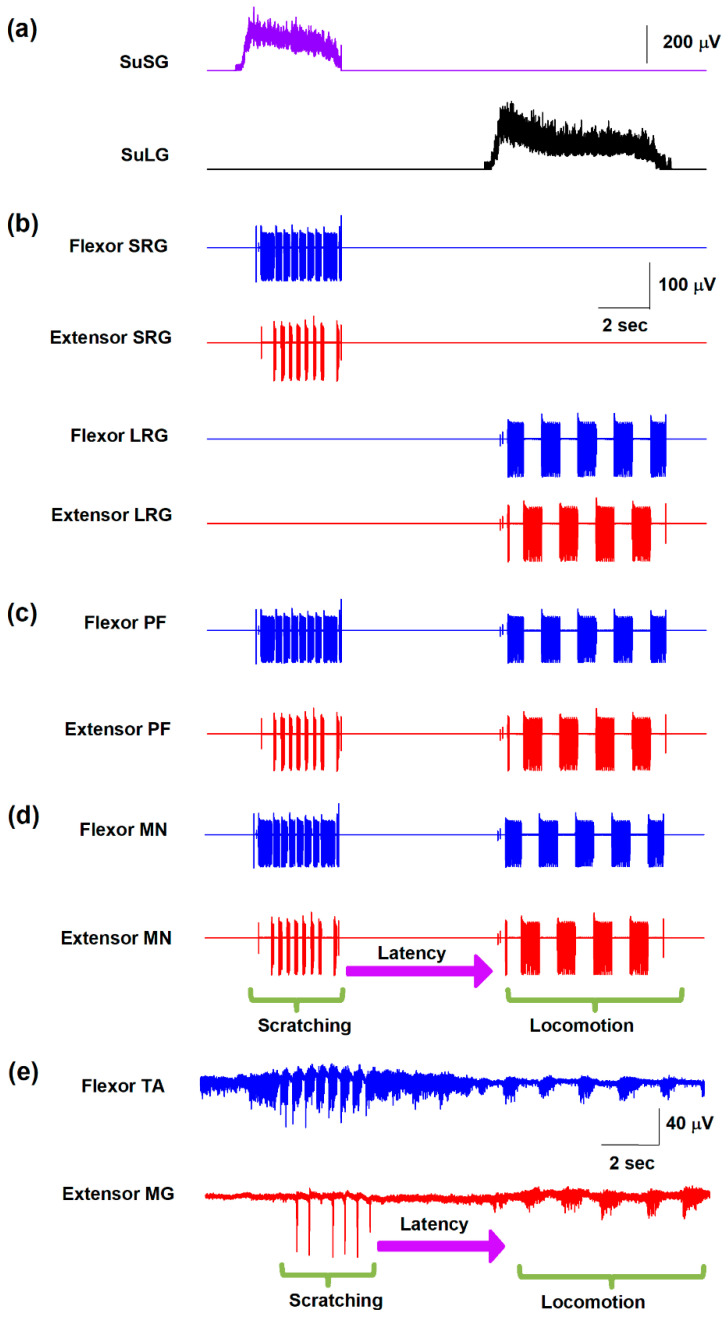
Numerical results of a simulated scratching and post-scratching locomotion episode from the model illustrated in Figure 1. (**a**) Simulated activity in the supraspinal scratching generator (SuSG) and supraspinal locomotion generator (SuLG). (**b**) Simulated firing in the scratching rhythm generator (SRG) and locomotion rhythm generator (LRG). (**c**) Simulated firing in the pattern formation (PF) layer. (**d**) Simulated firing in the motoneurons (MNs). Flexor activity is indicated in blue and extensor activity in red. Same color code as in Figure 1. (**e**) Electroneurographic recordings of tibial anterior (TA) and medial gastrocnemius (MG) nerves during a post-scratching locomotion episode in the cat.

Despite their slow frequency discharge, SuSG exerts a potent inhibition towards their counterpart called the supraspinal locomotion generator (SuLG). This could be a possible cause for the short periods of scratching activation and the delay between scratching and locomotion. When the SuLG is released from inhibition, such neurons start firing. Then, this activation is directed to the LRG and PF layer to produce the onset of locomotion. Figure 2 shows the simulated electrical activity obtained from the numerical analysis of the model illustrated in Figure 1. Note the different profiles in firing activity produced by each central pattern generator network and the delay in post-scratching locomotion, which is similar to experimental recordings of flexor and extensor electroneurograms obtained in the cat (Figure 2e).

There is a long gap or latency (of several seconds) between scratching and locomotion of 6.8 ± 3.9 s in our model and 6.2 ± 3.5 s in the experimental study. According to our model, a possible explanation for this long latency between scratching and locomotion could be the time required to switch from one motor task to another, which is commanded by the *φ* parameter in the SuSG half center. This *φ* parameter in the Morris–Lecar equations is interpreted as a factor of temporal dynamics in the neuronal electrical activity of SuSG and SuLG [14,15]. Once SuSG reduces its electrical firing, the selected value of 0.0002 s^−1^ for the *φ* parameter allows a long latency of inactivity of 6.8 ± 3.9 s until the other SuLG half center starts to respond (see Figure 2a). Thus, the *φ* = 0.0002 s^−1^ parameter in the SuSG-SuLG network is commanding the temporal dynamics for the functional reconfiguration from scratching to locomotion in the CPG networks.

When the SuSG and SuLG populations increase their firing rates, it takes 0.98 s and 1.01 s to recruit neurons from SRG and LRG, respectively. These latencies also are commanded by the *φ* = 0.23 s^−1^ and *φ* = 0.22 s^−1^ parameters in the SRG and LRG half centers, respectively.

**Figure 3:**
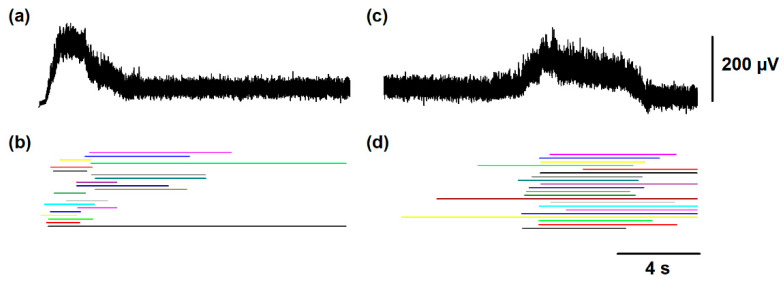
(**a**) Simulated activity in the supraspinal scratching generator (SuSG). (**b**) Raster of firing activity for those neurons in the SuSG network. The colors indicate the different trials of the numerical simulations with the model shown in Figure 1. The simulated slow potential in (**a**) was obtained from the firing activity in (**b**). (**c**,**d**) the same as (**a**,**b**) but for the supraspinal locomotion generator (SuLG).

**Figure 4:**
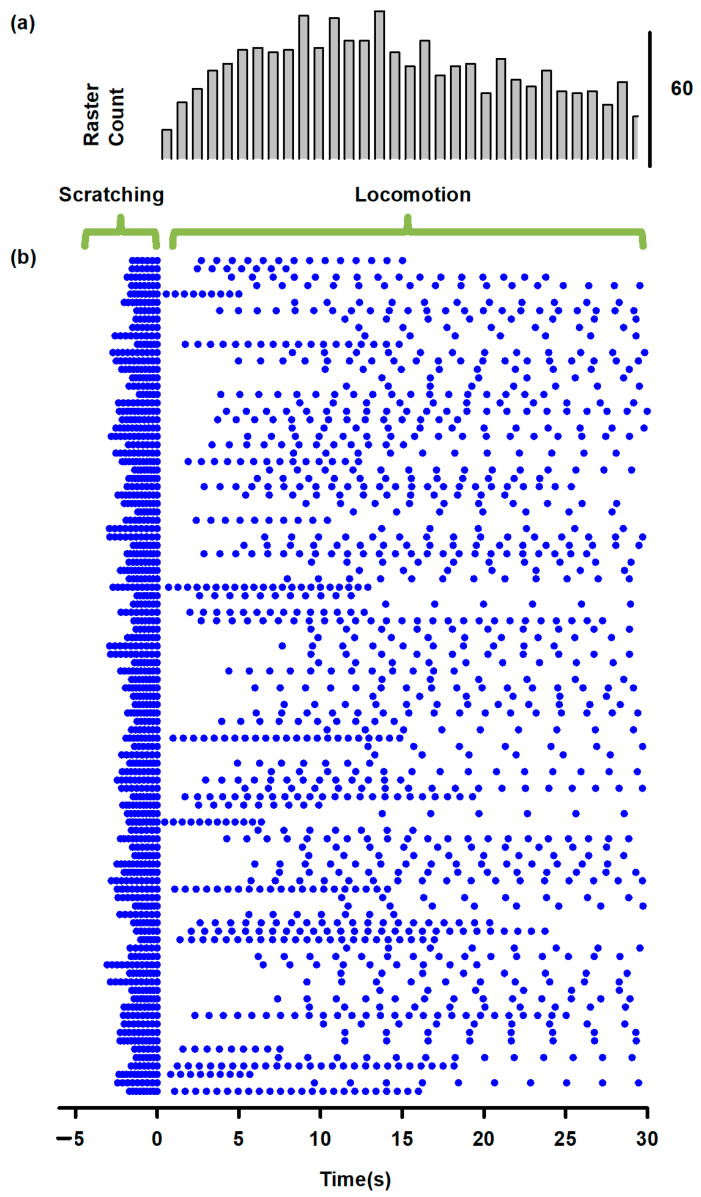
Numerical results of scratching and post-scratching locomotion obtained with the model illustrated in Figure 1. (**a**) Time distribution histogram of flexor bursts during the simulated scratching and post-scratching locomotion. (**b**) Raster display of 100 trials for the burst ending of the summed activity obtained from the entire simulated flexor motoneuron group, during scratching and post-scratching locomotion. Every horizontally aligned set of points in the y-axis represent a single simulation (a trial) of scratching followed by post-scratching locomotion. These numerical results are consistent with previous experimental results [13], specifically because there is not a statistically significant difference between the post-scratching locomotion latency of 6.8 ± 3.9 s obtained from this figure and the latency found in the experiments of 6.2 ± 3.5 s. Other specific criteria to justify our theoretical-experimental comparisons are that the cycle duration for scratching and locomotion in our simulation (0.2 ± 0.04 s and 1.6 ± 0.7 s) were not statistically different from those cycle durations observed in the experiments (0.3 ± 0.09 s and 1.7 ± 0.6 s, respectively).

Figure 3a,c show how the slow electrical potentials produced by the SuSG and SuLG networks (illustrated in Figure 2a) were obtained. These slow potentials were obtained from the electrical activity of populations of excitatory and inhibitory neurons in the SuSG and SuLG networks, respectively. Figure 3b,d show a raster display for the firing activity of some neurons. Since these raster graphs were constructed by changing the variability among the *µ* and *v_L_* parameters, their time and phase of activation are highly variable. Therefore, the duration of their synaptic action to their respective rhythm generator layers (scratching and locomotion) is also highly variable, producing a wide range of scratching and post-scratching locomotion episodes as illustrated in Figure 4. We found that the firing patterns shown in Figure 4 are similar to the experimental results of post-scratching locomotion in our previous report [13].

The zero on the time axis of Figure 4 represents the ending of different scratching episodes, indicated by the beginning of the arrow in Figure 2d. With this zero, we can compare the latencies between the end of each simulated scratching and the beginning of locomotion.

The high level of variability observed in Figure 3 and Figure 4 comes from the random variability assigned to the V_L_ and µ parameters (note the standard deviation of these parameters in Table 1). We adjusted the standard deviation of these parameters to obtain a random effect on the scratching duration, latency duration, and the scratching and post-scratching locomotion frequencies illustrated in Figure 4. This variability in our numerical results resembles the variability observed in previous experiments [13].

We calculated the mean post-scratching locomotion latency, i.e., the mean latency from the end of the scratching episodes to the beginning of post-scratching locomotion in Figure 4. We obtained a mean post-scratching locomotion latency of 6.8 ± 3.9 s, which is similar to the latency found in the experimental study [13] of 6.2 ± 3.5 s. We did not find statistically significant differences between these theoretical and experimental values (*p* > 0.5, Student’s *t*-test).

We also calculated the mean cycle duration of the firing activity in our numerical model during scratching and post-scratching locomotion. We found a mean cycle duration for scratching of 0.2 ± 0.04 s and a mean cycle duration for post-scratching locomotion of 1.6 ± 0.7 s. These values are also similar to those obtained in the experimental study [13], 0.3 ± 0.09 s of cycle duration for scratching and 1.7 ± 0.6 s of cycle duration for post-scratching locomotion. We did not find statistically significant differences between these theoretical and experimental values (*p* > 0.5, Student’s *t*-test).

**Figure 5:**
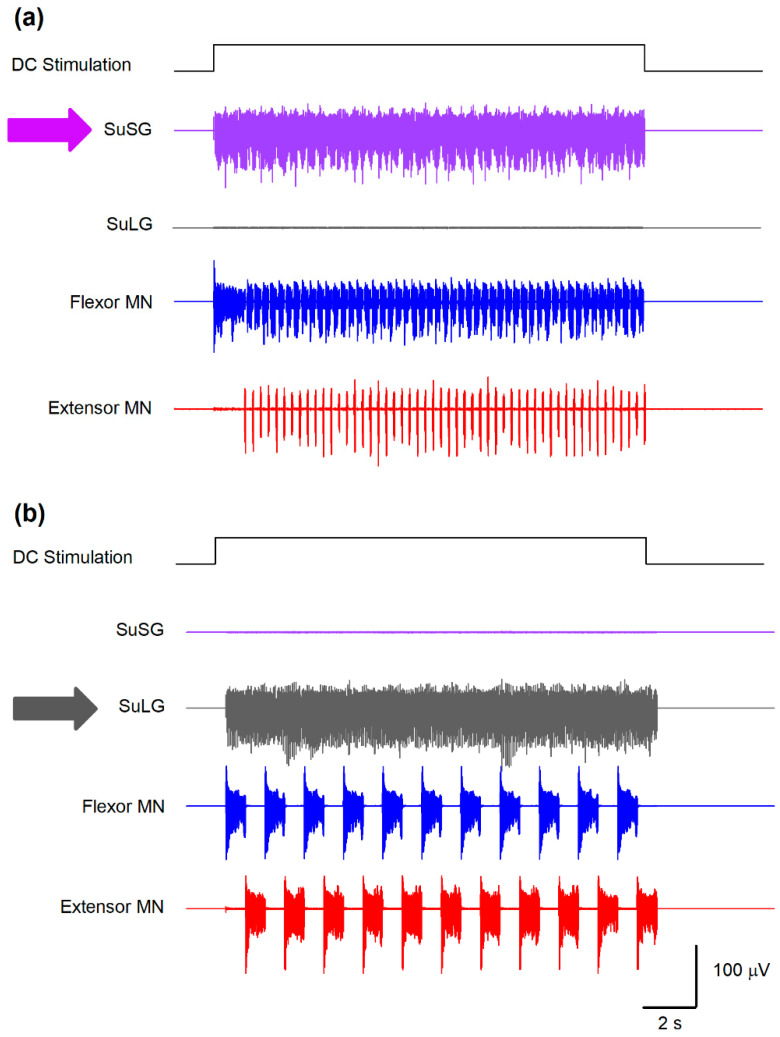
Predictions obtained from the model illustrated in Figure 1. (**a**) A selective sustained electrical stimulation of the SuSG, with a lesion in the SuLG region, will produce scratching episodes with an absence of post-scratching locomotion. (**b**) Conversely, a selective sustained electrical stimulation of the SuLG, with a lesion of the SuSG region, will only produce locomotion episodes with an absence of scratching. These predictions could be verified by the selective electrical stimulation to the brainstem at the obex level in animals with a lesion of the mesencephalic locomotor region (MLR) or selective stimulation of the MLR in animals with a lesion at the obex level. The direct current (DC) stimulation pulse consisted of 47 μA/cm^2^ and 16.6 s.

Based on the analysis of the Morris–Lecar model by Rinzel and Ermentrout [14], both the *μ* and *ε* parameters govern the calcium-dependent channel dynamics, which reproduces a bursting behavior. Specifically, *μ* is determined by the ratio between the surface of the cell and the total calcium near the membrane. The parameter *ε* is the product of the calcium degradation rate and the ratio of free to total calcium. Both parameters control the duration of the bursts and the duty cycle between flexor-extensor cycles for scratching and locomotion. As explained in Figure 2, the *φ* parameter in the SuSG-SuLG network is commanding the temporal dynamics for the functional reconfiguration from scratching to locomotion in the CPG networks and allows the long delay between scratching and locomotion. Therefore, systematic variations in some of these parameters affect the burst duration, burst frequency, and delay between scratching and locomotion and would lead to a testable hypothesis. For instance, the application of direct current (DC) stimulation of 47 μA/cm^2^ (during 16.6 s) to the SuSG and eliminating the parameters for the SuLG induces only a scratching behavior as illustrated in Figure 5a.

We also explored two predictions from this model to test its validity. The first prediction shown in Figure 5a suggests that a lesion of the SuLG and the direct current (DC) stimulation to the SuSG will only produce scratching episodes in the cat spinal cord. Note how the fast alternating activity of flexor and extensor motoneurons (blue and red traces in Figure 5a) during scratching occurs during the DC stimulation. In contrast, the second prediction shown in Figure 5b reveals that a lesion of the SuSG and the DC stimulation of SuLG will only evoke locomotion in the cat spinal cord. Again, note the slow alternating activity of flexor and extensor motoneurons (blue and red traces in Figure 5b) typical of locomotion.

## 4. Discussion

We developed a CPG computational model to explain a possible mechanism for the switching between scratching to post-scratching locomotion in the cat. In this model, we propose that there is a half center array presumably located at the supraspinal level. It is formed by one group associated with the onset of scratching and one related to the beginning of locomotion. Each group provides input to a spinal rhythm generator producing scratching and another producing locomotion. Both rhythm generators have an output to a common pattern formation. 

### 4.1. Descending Drives to Produce Different Motor Behaviors

Our model is consistent with previous studies suggesting that different descending pathways from supraspinal structures mediate distinct motor tasks. For instance, in the hatching tadpole, some motoneurons and inhibitory interneurons are active during swimming and struggling. It was suggested that the switching could be commanded by descending inputs [19]. Furthermore, a small region of the caudal hindbrain and rostral spinal cord was described that is sufficient to generate prolonged swimming in response to a brief stimulus [20]. As in our model, the neurons located in this region make reciprocal excitatory connections with each other and have ipsilateral descending axons and long-duration action potentials. Interestingly, these neurons are weakly active or even silent during struggling. On the other hand, different types of descending neurons located in the same regions (hindbrain and rostral spinal cord) are only active during a struggle but have a shorter duration action potential and fire repetitively when depolarized by current injection [19].

Experimental evidence that rhythmic movements can be induced by electrical stimulation of the mesencephalic locomotor region (MLR) and ventromedial medulla (VMM) [21,22] inspired the schematic design of our model. In a similar way, there are command neurons that initiate scratching in the brainstem at the obex level [23]. With this in mind, we suggest that descending inputs from the MLR or VMM could resemble descending inputs from the SuLG in our model. On the other hand, descending inputs from the same area in the midbrain could also generate the transition from one rhythmic motor task to another. For instance, there is experimental evidence that low-intensity electrical stimulation to the MLR in the salamander induces a walking gait. In contrast, higher stimulation intensities cause a rapid switch to swimming [24]. A limitation of our study is that we did not explore this possibility in our model.

However, other studies suggest that the descending inputs are unnecessary to allow switching between rhythmic motor tasks. For example, it was found that simultaneous and segmental swim-evoking and scratch-evoking stimulation in the turtle preparation results in interactions of scratch inputs with subthreshold swim inputs. It produces normal swimming, acceleration of the swimming rhythm, scratch-swim hybrid cycles, or complete cessation of the rhythm [6]. It was suggested that switching from one motor task to another underlies a functional reconfiguration of the network. That is, one motor task does not overlap the next. This reconfiguration takes place by recruiting neurons, changing the properties of active neurons, or silencing them. This involves continuous shifts in the set of active excitatory and inhibitory interneurons, as reviewed in [25].

The switch from one rhythmic motor task to another has also been explained by using robot models without the assumption of descending inputs [26]. In this context, it is possible that our model could be adapted to design bioinspired robots that could change from one rhythmic task to another but including the action of descending inputs to control the transition between such rhythmic motor tasks.

### 4.2. Multifunctional Interneurons 

In the present study, we proposed two different rhythm generators (for scratching and locomotion) that share the same pattern formation network (Figure 1). This suggests that such a pattern formation network serves as a multifunctional set of interneurons to produce scratching or post-scratching locomotion. This theoretical possibility sheds light on the open question of whether the locomotion and scratching CPG architectures constitute separate spinal cord networks dedicated to producing only one behavior or, in the opposite case, they conform to only one neuronal group of shared elements. Future experiments will be necessary to examine our hypothesis in detail.

Consistent with our proposal of a shared pattern formation and two different rhythm generators, some reports have shown that other neuronal populations can be used to generate a specific motor task. An example of this is seen in a report on the locus, in which the motoneurons innervating bifunctional muscles are active during walking and flight. Still, these patterns are produced by two distinct interneuronal networks [27]. All flight interneurons are inactive or tonically inhibited during walking. Additionally, all the interneurons that are modulated during walking are inactive, inhibited, or only weakly modulated during flight. Similar results were found in the zebrafish larva; by using calcium imaging, it was found that two different sets of interneurons drive swimming and escape tasks [28]. Additional evidence supports the idea of multifunctional interneurons and circuitry reconfiguration during the performance of different motor behaviors [29,30,31,32,33].

Different studies suggest that vertebrate limb movements are produced by spinal cord networks that share interneuron components. The turtle spinal cord can generate three distinct forms of scratching in three adjacent body regions when mechanical stimulation is applied [34]. Some neurons may contribute to the generation of the hip rhythm for all three forms of scratching, strengthening the case that vertebrate pattern-generating circuitry for distinct behaviors can be overlapping [35]. Along with these findings, many spinal interneurons are rhythmically active during ipsilateral and contralateral scratching [7,35]. Similarly, many spinal neurons are active during contralateral fictive scratching, ipsilateral fictive hindlimb withdrawal [8], or during scratching, fictive forward swimming, and fictive hindlimb withdrawal [7]. At the same time, a minority of spinal neurons are activated during scratching but silenced during swimming [7]. This last observation shows that even though there are neurons activated in scratching and other motor tasks, not all of the network generating scratching and locomotion is shared. In this context, evidence in the cat indicates a different regulation of the cycle period, phase durations, and phase transitions during fictive locomotion compared to scratching, providing evidence of specialized rhythm-generating mechanisms in each motor task [36]. In contrast, other investigations stated a similarity of effects from extensor group I input on the rhythmicity during scratching and locomotion, supporting the idea of a shared network [37].

### 4.3. Neuronal Topology

The rationale for using only 10 neurons per half-center group (100 neurons in total), as well as the divergence between groups of 20% (300 synaptic connections in total) is that these numbers were consistent with the number of neurons and synaptic connections employed in our previous model for scratching in cats [15]. Furthermore, we explored the minimal size of the total neuronal population to optimize our simulations, given that we simulated the neuronal electrical activity up to 35 s and employed several parameters (see Table 1). The selection of these 100 neurons and 300 synaptic connections allowed stable simulations according to our computational resources. However, in future studies, it will be necessary to examine other topologies of convergence and divergence with a more significant number of neurons and synaptic connections, as in the modeling of scratching CPG networks in the turtle [38].

### 4.4. Predictions

Several predictions could emerge from our model and could be tested in future experiments. First, the existence of two mutually inhibited bursting neuronal populations (termed here SuSG and SuLG) possibly located at the lower brainstem (SuSG) and MLR (SuLG) could be tested experimentally. The existence of these bursting neurons is supported by a previous experimental study from our laboratory [23]. The presence of bulbar interneurons in the obex region that exhibit on-off and off-on firing patterns before, during, and after fictive scratching was documented [23]. It could be interesting to verify through experiments the role of these bursting neurons during fictive scratching and post-scratching locomotion and determine if these neurons have any synaptic connections with bursting neurons at the level of the MLR. The simultaneous microstimulation and recording of bulbar and MLR neurons with bursting behavior could help to elucidate this possibility. Second, because specific values of parameters *ε*, *μ*, and *φ* (Table 1) are required to recreate the experimental findings, such numerical values may reflect the existence of particular intrinsic properties of supraspinal and spinal neurons. In this context, a detailed characterization of SRG, LRG, and PF neurons will be necessary to compare them with the model. Biologically, cells belonging to the supraspinal structures may have different channel densities or even different nonlinear dynamics regarding the various ionic channels producing the action potentials. Hence, this could be explored experimentally with the analysis of the intrinsic properties of these types of cells. Third, as Figure 5 illustrates, our model predicts that a lesion of the SuLG (i.e., the MLR) and the concomitant DC-stimulation to the SuSG (obex bulbar region) will only produce scratching episodes in the cat spinal cord. Conversely, a lesion of the SuSG (i.e., obex in the bulbar region) and the DC-stimulation of the SuLG (MLR) will only evoke locomotion in the cat spinal cord. These predictions derived from our model could be examined in the cat or other animal models.

### 4.5. Lateralization

Lateralization in the brain is the tendency for some neural processes to be specialized to one side of the brain or the other. Left-right asymmetries in the brain and in behavior have been described in diverse motor, sensory, cognitive, and affective conditions [39]. This lateralization is widespread among vertebrates and invertebrates [39] and has been credited for individuals outperforming their non-lateralized counterparts, increasing individual efficiency, and suggesting a solid contribution to biological fitness [40,41]. Brain asymmetries might prevent unnecessary duplication of neural circuitry, reduce interference between functions, and avoid the simultaneous initiation of incompatible responses by permitting only one hemisphere to control the responses [41]. In this context, scratching is a lateralized motor behavior that is generated by CPG circuits either active on the left or right side of the spinal cord. In contrast, the bilateral and alternating motor output between the two sides of the spinal cord during locomotion is mainly due to the inhibitory and excitatory balance over the midline. A limitation of our model is that we did not examine the differences in the lateralized behavior of scratching and locomotion. However, in future modeling studies, it will be interesting to investigate these critical differences. For example, it will be interesting to examine whether a bilateral distribution of SuSG-SuLG networks could allow left or right-lateralized scratching behavior. On the other hand, it is tempting to speculate that the symmetric organization of the locomotion CPG networks could be evolved to reduce interferences with the asymmetric scratching CPGs.

## 5. Conclusions

We conclude that the CPG model proposed in this study reproduces the main features of scratching and post-scratching locomotion. In particular, we conclude that these findings show how the transition of two rhythmic movements could be mediated by information exchange between their CPG circuits through routes converging in a common pattern formation layer. This integrated organization may provide flexible and effective connectivity despite the rigidity of the anatomical connections in the spinal cord circuitry.

## Figures and Tables

**Table 1:** Parameters used in the numerical simulations.

Parameter	Value		Parameter	Value	
*g_Ca_*	4.0 mS/cm^2^		*ε*	SuSG-SuLG	1.75 × 10^–2^ s^−1^
*g_K_*	8.0 mS/cm^2^			LRG	4 × 10^–4^ s^−1^
*g_L_*	2.0 mS/cm^2^			SRG	1.75 × 10^–3^ s^−1^
*g_KCa_*	0.25 mS/cm^2^			PF	4 × 10^–4^ s^−1^
*V_Ca_*	120 mV			MN	4 × 10^–4^ s^−1^
*V_K_*	−84.0 mV		*µ*	All other neurons	0.2 ± 0.05
*V_L_*	−60.0 ± 6 mV			SRG_Flexor	0.1 ± 0.01
*V*_1_	1.2 mV			SRG_Extensor	0.178 ± 0.02
*V*_2_	−18 mV		*I_App_*	45 mA/cm^2^ for SuSG and SuLG; 43.8 mA/cm^2^ for every other neuron	
*W*_1_	12 mV		*α*	0.1 ms^−1^	
*W*_2_	17.4 mV		*β*	0.2 ms^−1^	
*φ*	SuSG SuLG	0.0002 s^−1^	*E_S_*	0 mV (Excitatory); −80 mV (Inhibitory)	
	LRG	0.22 s^−1^	*g^syn^*	0.1 ± 0.01 mS/cm^2^	
	SRG	0.23 s^−1^			
	All other neurons	0.20 s^−1^	*Ca*_0_	10 mM	

## Data Availability

The authors confirm that all data underlying the findings are fully available without restriction. All relevant data are within the paper.
